# Development and Psychometric Evaluation of the Father Engagement Questionnaire

**DOI:** 10.1007/s10826-018-1195-0

**Published:** 2018-07-20

**Authors:** Yixin Jiang, Lucy A. Tully, Matthew T. Burn, Patrycja Piotrowska, Daniel A. J. Collins, Caroline Moul, Paul J. Frick, David J. Hawes, Eva R. Kimonis, Rhoshel K. Lenroot, Vicki Anderson, Mark R. Dadds

**Affiliations:** 1 School of Psychology, University of Sydney, Sydney, Australia; 2 Learning Sciences Institute of Australia, Australian Catholic University, Brisbane City, QLD 4000 Australia; 3 Department of Psychology, Louisiana State University, Baton Rouge, LA 70803 USA; 4 School of Psychology, University of New South Wales, Sydney, NSW 2052 Australia; 5 School of Psychiatry, Faculty of Medicine, University of New South Wales, Sydney, NSW 2052 Australia; 6 Royal Children’s Hospital, Murdoch Children’s Research Institute, Parkville, VIC 3052 Australia; 7 Departments of Psychology & Pediatrics, University of Melbourne, Melbourne, VIC 3010 Australia

**Keywords:** Parenting programs, Father engagement, Measure development, Assessment, Parenting

## Abstract

While there has been increasing interest in promoting father engagement in parenting interventions for child wellbeing, both research and practice endeavors have been hindered by a lack of a measure of father engagement practices. This paper reports the development and evaluation of a comprehensive, practitioner-report measure of father engagement practices–—the *Father Engagement Questionnaire* (FEQ). Practitioners (*N* = 589; 84.5% females; mean age = 38.56) involved in delivering parenting interventions in Australia completed the FEQ, along with background demographics and questions regarding their own and organization’s practice. A separate sample of 28 practitioners completed the FEQ twice, with a two-week interim, to assess test–retest stability of the measure. Exploratory factor analysis revealed five factors corresponding to the measure’s five intended content areas: Confidence in Working with Fathers, Competence in Using Engagement Strategies, Perceived Effectiveness of Engagement Strategies, Frequency of Strategy Use, and Organizational Practices for Father Engagement. Each of these scales demonstrated adequate internal consistency reliability and test–retest stability. As the five scales appear to be related but distinct, it is recommended that the FEQ is used as a multidimensional measure of father engagement. In terms of predictive validity, higher scores on the Confidence in Working with Fathers, Frequency of Strategy Use, and Organizational Practices for Father Engagement scales were associated with a higher likelihood of practitioner-reported father attendance. The results provide support for adequate psychometric properties of the FEQ as a research and clinical tool for assessing and monitoring father engagement practices.

## Introduction

Fathers (i.e., male caregivers) play a vital role in the lives of their children, conferring unique protective and risk processes associated with development (Campbell et al. 2014; Lamb 2004). In the last decade, there has been increasing research and practice focus on the importance of father participation and engagement in parenting interventions for child wellbeing. Evidence-based parenting interventions, which are delivered in diverse professional settings, have been shown to have both immediate and long-term positive effects on parent and child outcomes (Kaminski and Claussen 2017; Lundahl et al. 2008; Nores and Barnett 2010) and father participation improves short-term outcomes for parenting and child behavior (Lundahl et al. 2008). However, the rates of father attendance and engagement in these programs have been found to be very low (see Panter-Brick et al. 2014, for a review). Key factors that appear to contribute to the low levels of father engagement include practitioners’ competencies in engaging fathers, and organizational levels of support for father-inclusive practice (Tully et al. 2017). However, there are no existing measures of practitioners’ and organizational father engagement practices in the context of delivering parenting interventions.

Research has identified that low levels of practitioners’ skills and knowledge can be a barrier to father engagement. For example, McBride and Rane (2001) found that low rates of father participation in Head Start programs in America could be attributed to low levels of practitioner knowledge and skills about engaging fathers. Similarly, McAllister et al. (2004) identified staff attitudes (e.g., gender stereotyping), experiences (e.g., with their own fathers and other men), and resources (e.g., staff training) as barriers to father involvement. Moreover, there is evidence that training practitioners in skills to enhance father engagement is associated with greater practitioner competence as well as increased rates of father engagement (Scourfield et al. 2012). However, few practitioners participate in father engagement training (Tully et al. 2017) and the need to develop and disseminate training programs has been highlighted (Zanoni et al. 2013). Given the need to assess practitioners’ skills and competence in father engagement, particularly in the context of evaluating outcomes of practitioner training programs, it is important to develop a more psychometrically valid measure of practitioners’ father engagement practice.

There is limited research on which specific practitioner qualities and competencies contribute to successful father engagement practice. Scourfield et al. (2012) found that increased self-efficacy or confidence of social workers after participating in a two-day father engagement training course was related to increased caseload engagement of low risk fathers in a child protection context. This suggests that practitioners’ confidence in working with fathers may be linked to their success in engaging fathers. Indeed, confidence is viewed as the cognitive precursor to implementation/practice of specific skills (Turner et al. 2011). The domains of confidence assessed by Scourfield et al. (2012) included motivating fathers without increasing resistance, engaging men who appear hostile or aggressive, developing an open and honest relationship with fathers, highlighting fathers’ strengths and positive qualities, helping fathers understand their behavior and role, and assessing fathers’ parenting risk to their children. While intended to be relevant to child protection work, these domains of confidence (with the exception of assessing risk) may also be relevant to engaging fathers in parenting interventions for child wellbeing more generally.

Practitioners’ confidence by itself may be insufficient for promoting father engagement practice; it is also important to take into account practitioners’ levels of competencies with respect to use of specific skills in engaging fathers. In a survey of 210 practitioners who delivered parenting interventions, Tully et al. (2018) found that two-thirds reported being confident in working with fathers. However, when confidence levels were considered together with practitioners’ reports of frequency of using specific father engagement strategies (together referred to as “competencies”), only one-quarter identified themselves as high in competence. Therefore, it is important to assess practitioners’ frequency of use of skills and competence in specific father engagement strategies.

In addition to practitioner confidence and competencies, organizational culture and practices should also be evaluated and addressed. Through surveys with practitioners, researchers have identified some common organizational barriers to father participation and engagement including: a culture of devaluing father involvement and/or not engaging the whole family (Lazar et al. 1991; McBride and Rane 1996; Potter and Carpenter 2008); fathers’ lack of awareness of the service due to advertising targeted to mothers only (Bayley et al. 2009); mother-oriented program delivery and content (Bayley et al. 2009; McBride et al. 2017); inflexible service hours (Bayley et al. 2009; McBride et al. 2017); and a general lack of organizational support and policy for father-inclusive practice (Bayley et al. 2009). Based on these organizational barriers, researchers have made various practice recommendations such as advertising that a service is for fathers, as well as mothers; offering sessions outside normal work hours (Bayley et al. 2009; McBride et al. 2017; Tully et al. 2018); obtaining assessment data from fathers as well as mothers; and emphasizing the importance of father attendance at intake (Tully et al. 2018). Glynn and Dale (2015) found that practitioners regarded organizational philosophy as one of the three most important factors to father engagement (along with practitioner qualities, and intervention content), and that this factor is perceived as highly amenable to change.

There is preliminary evidence to suggest that practitioner competencies and organizational practices are associated with father attendance rates. As already mentioned, Scourfield et al. (2012) found that a training program focusing on changing practitioners’ confidence led to increased rates of father engagement. Similarly, in a survey of practitioners delivering parenting interventions, Tully et al. (2018) found that practitioners’ competence predicted ratings of fathers often (as opposed to rarely) attend sessions. This study also found that practitioner-reported levels of organizational support for father-inclusive practice predicted a greater likelihood of fathers attending.

There are a few existing measures that assess constructs related to father engagement practice. Two measures—The Role of the Father Questionnaire (Palkovitz 1984) and the Attitudes Toward Father Involvement Scale (ATFI; Garinger and McBride 1995)—assess attitudes and beliefs about fathers and the role of fathering. However, these are neither specifically designed for practitioner-report, nor do they assess practitioners’ competencies, which are likely to be more proximally linked to father engagement practice than attitudes and beliefs. Another measure, called the Dakota Father Friendly Assessment Tool (White et al. 2011), assesses staff perceptions about their own attitudes and behavior, as well as their organization’s attitudes and behavior regarding father involvement in early childhood settings, but it does not explicitly assess practitioners’ competencies toward specific father engagement practices. The items in the measure also tend to be contextually specific (e.g., “Staff should involve fathers in parent-teacher meetings” and “Staff recruit fathers to parent advisory board, etc.”), as opposed to having broad relevance to a diverse range of parenting interventions for child wellbeing. Finally, Scourfield et al. (2012) presented a measure of practitioner self-efficacy in relation to child protection work with fathers but it neither assesses specific competencies nor organizational practices for engaging fathers, and is not relevant for practitioners delivering parenting interventions. Therefore, existing measures tend to have an attitudinal focus, are narrow in scope (e.g., self-efficacy), and developed for a specific context of service delivery.

The purpose of this paper was to evaluate the psychometric properties (i.e., internal structure, internal consistency reliability, test–retest stability, and predictive validity) of the FEQ. It was hypothesized that exploratory factor analysis would reveal a five-factor structure, corresponding to the five content areas of the questionnaire: confidence in working with fathers; competence, perceived effectiveness, and frequency of using the father engagement strategies; and organizational practices for father engagement. The factors were expected to be positively intercorrelated, and demonstrate both internal consistency and test–retest stability. In the absence of other existing measures of father engagement practice in parenting interventions, we examined the predictive validity of the FEQ scales, which were expected to predict practitioner-reported father attendance rates in parenting sessions.

## Method

### Participants

The main sample (Sample 1) consisted of 589 practitioners who worked in services or organizations that delivered parenting programs in Australia. They were recruited as part of a study examining the effectiveness of a father engagement training program, *Engaging Fathers in Parenting Programs: A National Training Program for Practitioners*, which was delivered either face-to-face or online. Of the sample, 85.7% reported that they worked directly with families, 4.4% were support staff (e.g., administrative staff and managers), and 8.8% reported they did not currently work with families but had done so previously. Furthermore, 84.5% were female and 14.5% were male (0.8% did not indicate their gender). The mean age was 38.56 (*SD* *=* 11.27). The dominant profession was psychologist (38.7%), followed by social worker (16.5%), and, thirdly, family support worker (9.4%). The remaining professions included counselor, caseworker, nurse, psychiatrist, general practitioner, occupational therapist, educator/teacher, family dispute resolution practitioner, director/manager, and administrative worker. Of the sample, 42.4% indicated that they worked in a non-government organization, 32.0% in a child and family mental health service or other government organization, 15.0% in private practice, 4.7% in a university-based clinic, and 5.1% indicated ‘other organization’. The mean years of experience working with families was 8.89 (*SD* = 7.95, range = 0–40), and the majority (80.5%) had not received previous training in father engagement.

### Procedure

All participants in Sample 1 completed all measures prior to receiving the father engagement training intervention. They completed the measures using either traditional paper-and-pencil versions (if they attended the training in person) or online, administered using Qualtrics^TM^ online survey software (if they participated in the online training). No differences in results were expected based on mode of administration, as previous research has established the psychometric equivalence of traditional paper-and-pencil and online versions of self-report questionnaires (e.g., Riva et al. 2003). The current study included only the pre-training data. The questionnaire took approximately 15 min to complete, and participants did not receive any incentives.

A separate sample of practitioners (*N* = 32; Sample 2), who were not involved in the father engagement training program, were recruited to complete the FEQ twice to assess the test–retest stability. The second questionnaire was completed approximately two weeks (mean = 14 days; range = 14–25 days) after the first completion. To ensure a high rate of completion on the second testing occasion, participants were given a $20 gift voucher to thank them for their time. Of the 32 practitioners who completed the first questionnaire, 28 (87.5%) (25 females and 3 males) completed the second questionnaire. These practitioners had a mean age of 40.75 years (*SD* = 15.20), and on average had 9.5 years of experience (*SD* = 6.18, range = 1–25) working with families. They worked primarily as a psychologist (46.4%) or social worker (21.4%). The practitioners were employed variously in a non-government organization (46.4%), a child and family mental health service or other government organization (25.0%), a university-based clinic (14.3%), private practice (10.7%), or other (3.5%). The majority (82.1%) had not previously participated in specific training for working with or engaging fathers. The study (including Sample 1 and Sample 2) was approved by the Human Research Ethics Committee at the University of Sydney. All participants read a Participant Information Statement and gave their consent prior to completing the measures: online participants indicated their consent by clicking a box and face-to-face participants signed a consent form.

### Measures

#### Father Engagement Questionnaire

The Father Engagement Questionnaire (FEQ) was developed by a team of researchers and clinicians at the University of Sydney. The questionnaire items were developed through a review of the literature related to father engagement, including barriers to participation, practitioner competencies, and potential father engagement strategies at the practitioner and organizational levels; and in consultation with a team of 10 researchers and clinical psychologists with extensive experience in delivering parenting interventions with families. The items (in both paper-and-pencil and computerized online format) were then pilot tested with a small convenience sample of 30 researchers and practitioners. Based on feedback from the pilot test, the items were then revised to improve clarity in wording before inclusion in the questionnaire. After pilot testing, this questionnaire contained 49 items that assessed 5 content areas.

For the first content area, a set of 15 items asked practitioners to rate how confident they felt regarding various aspects of working with fathers. These items included process issues (e.g., eliciting fathers’ expectations of treatment and goals, and understanding fathers’ needs), client vulnerabilities (e.g., working with separated/divorced parents), knowledge of the literature about father-child relationships, and encouraging their team/service/organization to use father-inclusive practices and policies. Practitioners rated all items on a Likert-type scale from *not at all confident* (1) to *extremely confident* (5).

The second, third, and fourth content areas assessed practitioners’ ratings of perceived effectiveness of, competence in using, and frequency of implementing 10 specific father engagement strategies respectively (e.g., listening to fathers and exploring barriers to engagement, and directly inviting fathers who are reluctant to attend). The 10 items regarding perceived effectiveness asked practitioners to rate each of the strategies using the prompt, “To what extent do you believe the following strategies are effective for increasing the engagement of fathers?” using a Likert-type scale from *not at all effective* (1) to *extremely effective* (5). The 10 items regarding competence asked practitioners to rate the same strategies using the prompt, “To what extent do you feel competent to implement the following strategies with fathers?” using a Likert-type scale from *not at all competent* (1) to *extremely competent* (5). The 10 items regarding frequency of strategy use asked practitioners to rate the strategies using the prompt, “Over the last two months, to what extent have you used the following strategies when working with fathers and families?” using a Likert-type scale from *never* (1) to *always* (5).

Lastly, pertaining to the fifth content area, practitioners were asked to rate four items regarding the degree to which their service/program uses father engagement strategies. Each item contained the stem, “How often does your service/program use the following strategies to engage fathers?” Practitioners then rated four strategies—emphasizing the importance of father attendance at intake, offering sessions outside work hours, advertising that the program/treatment is for fathers as well as mothers, and obtaining data from fathers as well as mothers—on a Likert-type scale from *never* (1) to *always* (5).

#### Current Rates of Father Attendance

Practitioners were asked to indicate which statement best reflects their work with fathers over the past two months: *fathers never attend sessions*, *fathers rarely attend sessions*, *fathers sometimes attend sessions*, *fathers often attend sessions*, and *fathers always attend sessions*. Given the small proportion of practitioners that indicated *fathers never attend sessions* (2.5%), this category was combined with *fathers rarely attend sessions* (27.4%) in subsequent analyses. Similarly, given the small proportion indicating *fathers always attend sessions* (3.9%), this category was combined with *fathers often attend sessions* (13.4%) in subsequent analyses. Of the sample, 38% indicated that *fathers sometimes attend sessions*. A small proportion (13.5%) of practitioners indicated that they had not worked with families in the past two months, and their responses regarding current rate of father attendance were excluded from subsequent analyses including this variable.

### Data Analyses

All analyses, with the exception of the test–retest stability, were conducted on Sample 1. Exploratory factor analyses (EFA) were conducted to examine the factor structure of the FEQ. The inclusion of items in the questionnaire was revised according to the results of the EFAs to obtain a clear solution that was also theoretically sound. To determine the number of factors to retain, we used parallel analysis (O’Connor 2000), Velicer’s Minimum Average Partial (MAP) test (Velicer et al. 2000), and Kaiser’s (1960) criterion, along with inspecting the screeplot. The scales based on the factors derived from the final EFA solution were then examined for internal consistency using Cronbach’s alpha reliability and test–retest stability using intraclass correlations (for Sample 2). To examine predictive validity, multinomial logistic regression was conducted to examine the prediction of practitioner-reported father attendance rates using mean ratings on the FEQ scales as predictors. All analyses were performed in IBM SPSS Statistics Version 23.

## Results

### Factor Structure

An initial exploratory factor analysis (EFA) was conducted on all 49 items to examine the factor structure of the FEQ. Prior to performing the EFA, suitability of the data for factor analysis was assessed via inspection of the correlation matrix. Positive manifolds of medium to large intercorrelations were observed between items that were designed to assess the same domain. Correlations between items that were designed to assess different content areas tended to be smaller, especially between organizational practices for father engagement items and all other questionnaire items (most *r*’s < 0.30). The Kaiser-Meyer-Olkin value was 0.93, which is larger than the recommended minimum value of .60 (Kaiser 1970), and Bartlett’s Test of Sphericity (Bartlett 1954) reached statistical significance *χ*^2^ (1176) = 14875.36, *p* < 0.001, further supporting the factorability of the correlation matrix. Moreover, an inspection of the anti-image correlation matrix (AIC), which shows the measure of sampling adequacy (MSA) along the diagonal, revealed high MSA values for each item between 0.86 and 0.97 (juxtaposed by low off-diagonal values). This indicates that each of the 49 items is strongly correlated with the other items in the matrix. Together, these indicators support the factorability of the correlation matrix for all 49 items (Pett et al. 2003).

In the initial EFA [maximum likelihood (ML) with PROMAX rotation], potential solutions included: six factors (based on the Parallel Analysis), eight factors (based on screeplot, and Velicer’s MAP test), or nine factors (based on Kaiser’s criterion), accounting for between 58.67 and 65.70% of common variance. An inspection of the pattern matrix revealed cross-loadings of several items. One cross-loading item—“To what extent do you feel competent to implement the following strategies with fathers?—Listening to fathers and exploring their barriers to engagement”—was retained due to its face validity and reasonable sized primary factor loading (0.64) and small secondary loading (0.36). All other cross-loading items were removed. To maintain consistency across the questionnaire, any cross-loading items were removed in all instances where the item-content appeared. Two additional items—“How confident do you feel in the following?—Working with fathers with substance use issues” and “How confident do you feel in the following?—Working with fathers who have been violent or abusive”—were also removed given that they loaded separately on a ninth factor, suggesting theoretical divergence from the other confidence items. These two items appear to tap a narrow domain of work with fathers with specific vulnerabilities (e.g., fathers with substance use issues) and did not appear to be as relevant as the rest of the items to engaging fathers in broader settings. Overall, a total of 17 items were removed.

The EFA (ML; PROMAX rotation) was re-run on the remaining 32 items. In this second EFA, the potential solutions include four factors (based on parallel analysis), five factors (based on screeplot and Velicer’s MAP test), or six factors (based on Kaiser’s criterion), accounting for between 57.99 and 65.36% of common variance. Given that both the screeplot and Velicer’s MAP test suggested five factors, the EFA was then constrained to five factors. In this third solution, there was one remaining cross-loading item: “To what extent do you feel competent to implement the following strategies with fathers—Using a father inclusive approach, e.g. flexible service delivery, marketing to fathers.” This cross-loading item was removed, along with the corresponding items in the other two factors that referred to the same content. One other item—“How confident do you feel in the following?—Ability to remain neutral (not side with the mother or father)”—was also removed due to a low factor loading of 0.29. After removing these four items, a final EFA (ML with PROMAX rotation) constrained to five factors was performed. The results of this EFA are presented in Table 1.

**Table 1:** Factor loadings, item-to-total correlations, and communalities from an EFA (maximum likelihood with PROMAX rotation) constrained to five factors after removing cross-loading items (*N* = 589)

	Factor					Unrotated factor loading on first principle component	Corrected item-total *r*	
	1	2	3	4	5			*h* ^*2*^
*How confident do you feel in the following?*								
Dealing with resistance from fathers	0.90					0.61	0.57	0.62
Engaging fathers who are reluctant to attend	0.85					0.60	0.56	0.57
Managing conflict between myself and fathers	0.81					0.65	0.60	0.62
Managing distress from fathers	0.71					0.67	0.62	0.60
Communicating with fathers	0.57					0.64	0.58	0.52
Managing conflict between mothers and fathers	0.55					0.71	0.68	0.56
Understanding fathers’ needs	0.51					0.61	0.57	0.45
Eliciting fathers’ expectations of treatment and their goals	0.51					0.62	0.57	0.46
Working with separated/divorced parents	0.39					0.60	0.54	0.40
*Over the last two months, to what extent have you used the following strategies when working with fathers and families?*								
Exploring feelings underlying anger, hostility or blame when it arises		0.84				0.68	0.66	0.70
Managing conflict (practitioner-client and mother-father)		0.84				0.66	0.62	0.67
Listening reflectively and creating a shared understanding about both parents’ perspectives (even when they differ)		0.82				0.68	0.63	0.70
Negotiating shared goals and expectations (practitioner-father-mother)		0.80				0.65	0.60	0.67
Listening to fathers and exploring their barriers to engagement		0.72				0.67	0.64	0.63
*To what extent do you believe the following strategies are effective for increasing the engagement of fathers?*								
Exploring feelings underlying anger, hostility or blame when it arises			0.65			0.33	0.34	0.46
Managing conflict (practitioner-client and mother-father)			0.71			0.35	0.36	0.53
Listening reflectively and creating a shared understanding about both parents’ perspectives (even when they differ)			0.83			0.40	0.40	0.72
Negotiating shared goals and expectations (practitioner-father-mother)			0.80			0.36	0.33	0.63
Listening to fathers and exploring their barriers to engagement			0.72			0.37	0.37	0.52
*To what extent do you feel competent to implement the following strategies with fathers?*								
Exploring feelings underlying anger, hostility or blame when it arises				0.58		0.73	0.66	0.60
Managing conflict (practitioner-client and mother-father)				0.58		0.74	0.68	0.60
Listening reflectively and creating a shared understanding about both parents’ perspectives (even when they differ)				0.91		0.75	0.66	0.77
Negotiating shared goals and expectations (practitioner-father-mother)				0.81		0.75	0.67	0.70
Listening to fathers and exploring their barriers to engagement				0.72		0.68	0.60	0.59
*How often does your service/program use the following strategies to engage fathers?*								
Advertising that the program/treatment is for fathers as well as mothers					0.85	0.41	0.44	0.68
Obtaining data (about parenting or child behavior) from fathers as well as mothers					0.71	0.40	0.42	0.54
Emphasizing the importance of father attendance at intake					0.66	0.48	0.53	0.54
Offering sessions outside work hours to enable fathers to attend					0.48	0.33	0.36	0.27

As can be seen in Table 1, the five factors accounted for 65.38% of common variance. The first factor consisted of loadings from nine items tapping confidence with respect to different aspects of working with fathers, and was labeled *Confidence in Working with Fathers* (Confidence). The second factor consisted of loadings from five items tapping the frequency of using father engagement strategies, and was labeled *Frequency of Strategy Use*. The third factor consisted of loadings from five items related to the perceived effectiveness of father engagement strategies, and was labeled *Perceived Effectiveness of Engagement Strategies* (Perceived Effectiveness). The fourth factor consisted of loadings from five items tapping practitioner-reported competence in using father engagement strategies, and was labeled *Competence in Using Engagement Strategies* (Competence). Lastly, the fifth factor consisted of loadings from four items related to organization use of father engagement strategies, and was labeled *Organizational Practices for Father Engagement* (Organizational Practices). One item—“How often does your service/program use the following strategies to engage fathers?—Offering sessions outside work hours to enable fathers to attend”—had a low communality and factor loading. However, as there were only four items in total indexing Organizational Practices, this item was retained. All other items had acceptable communalities. Table 1 also shows the unrotated factor loadings on the first principle component and correct item-total correlations for all items. These factor loadings and correlations do not suggest that the items converge to a single overarching latent factor. Instead, a five-factor structure is clearer and more interpretable. Therefore, the final FEQ consisted of a total of 28 items, which formed five scales that corresponded with the five intended content areas.

The factor intercorrelations for the final questionnaire are presented in Table 2. Practitioners’ Confidence was highly correlated (*r* *=* 0.68, *p* < 0.001) with Competence, suggesting a high degree of construct overlap. Notably, there was also medium correlations between Frequency of Strategy Use and both Competence (*r* = 0.58 *p* < 0.001) and Confidence (*r* *=* 0.57, *p* *<* 0.001). Organizational Practices also had a medium correlation (*r* = 0.48, *p* < 0.001) with practitioners’ Frequency of Strategy Use. All other correlations between the factors were also significant and positive, but small-to-medium in size (*r* *=* 0.23 to 0.41), suggesting that the constructs underlying those factors were related but distinct. Given that none of the factor intercorrelations were exceedingly high, this supports the use of FEQ as composed of five separate scales tapping different but related aspects of father engagement.

**Table 2:** Intercorrelations, means, standard deviations, and internal reliabilities for all scales (*N* = 589)

	Pearson’s *r*					Mean (*SD*)	Cronbach’s α internal reliability
	1	2	3	4	5		
1 *Confidence in Working with Fathers*	1					3.00 (0.61)	0.90
*Father Engagement Strategies*							
2 Frequency of use	.57^*^	1				3.07 (0.90)	0.91
3 Perceived effectiveness	.27^*^	.23^*^	1			3.86 (0.62)	0.86
4 Competence	.68^*^	.58^*^	.41^*^	1		3.32 (0.66)	0.90
5 *Organizational practices for Father Engagement*	.35^*^	.48^*^	.18^*^	.36^*^	1	3.15 (0.97)	0.78

^*^p < 0.001

### Internal Consistency Reliability

The means, standard deviations, and internal consistency reliabilities are shown in Table 2. As can be seen in Table 2, the Cronbach’s alpha internal consistency reliabilities for all scales were acceptable, ranging from 0.78 to 0.91.

### Test–retest Stability

Sample two was used to examine test–retest stability of the 28 item FEQ. Intraclass correlation (ICC) estimates and their 95% confidence intervals were calculated based on a single-measure, absolute-agreement, and two-way mixed-effects model. Using Cichetti’s (1994) guidelines, the Competence scale demonstrated excellent ICC estimates of test–retest reliability: 0.98 (95% CI: 0.96, 0.99). Two of the scales demonstrated good estimates of test–retest reliability: Perceived Effectiveness (ICC = 0.77; 95% CI: 0.55, 0.89), and Organizational Practices (ICC = 0.70; 95% CI: 0.45, 0.85). The remaining two scales demonstrated moderate estimates of test–retest reliability: Confidence (ICC = 0.58; 95% CI: 0.27, 0.77) and Frequency of Strategy Use (ICC = 0.62; 95% CI: 0.29, 0.83). This suggests that the questionnaire scales have moderate to good test–retest stability over a two-week period.

### Predictive Validity

A multinomial logistic regression was conducted in which the FEQ scales (Confidence, Perceived Effectiveness, Competence, Frequency of Strategy Use, and Organizational Practices) were entered as predictors of practitioner-reported father attendance rate. The overall model was statistically significant, *χ*^2^ (10, *N* *=* 479) = 89.14, *p* < 0.001, accounting for between 17% (Cox and Snell Pseudo *R*^2^) and 19.4% (Nagelkerke Pseudo *R*^2^) of variance in father attendance response category, and correctly classified 53.7% of cases. As shown in Table 3, relative to “Fathers never/rarely attend sessions”, practitioners who reported higher levels of Confidence, Frequency of Strategy Use, and Organizational Practices were respectively 2.87, 2.14, and 2.04 times more likely to report that “Fathers often/always attend sessions”. Similarly, relative to “Fathers never/rarely attend sessions”, practitioners who reported higher levels of Frequency of Strategy Use and Organizational Practices were respectively 1.53 and 1.36 times more likely to report “Fathers sometimes attend sessions”. Conversely, and contrary to expectations, relative to "Fathers never/rarely attend sessions” practitioners who provided higher ratings of Perceived Effectiveness were 0.60 times less likely to report “Fathers sometimes attend sessions”. There were no other significant predictors of practitioner ratings of father attendance.

**Table 3:** Multinomial logistic regression predicting practitioners’ ratings of father attendance (*N* = 479)

Categories		*B*	S.E.	Sig.	Exp(*B*)	95% CI	
						Lower	Upper
Fathers often/always attend sessions	Intercept	−4.80	1.09	0.000			
	Confidence in working with Fathers	1.05	0.36	0.003	2.87	1.42	5.81
	Perceived effectiveness of engagement strategies	−0.38	0.26	0.145	0.68	0.41	1.14
	Competence in using engagement strategies	−0.65	0.35	0.063	0.52	0.26	1.04
	Frequency of strategy use	0.76	0.22	0.001	2.14	1.39	3.29
	Organizational practices for father engagement	0.71	0.17	0.000	2.04	1.46	2.85
Fathers sometimes attend sessions	Intercept	−0.17	0.81	0.834			
	Confidence in working with fathers	0.44	0.27	0.103	1.55	0.91	2.64
	Perceived efectiveness of engagement strategies	−0.52	0.19	0.008	0.60	0.41	0.87
	Competence in using engagement strategies	−0.31	0.26	0.229	0.73	0.44	1.22
	Frequency of strategy use	0.42	0.16	0.009	1.53	1.11	2.10
	Organizational practices for father engagement	0.31	0.13	0.016	1.36	1.06	1.75

The reference category is ‘Fathers never/rarely attend sessions

## Discussion

While increasing effort is being devoted to promoting father engagement in parenting interventions for child wellbeing, both research and practice endeavors are hindered by a lack of a suitable measure of father engagement practices. This paper reports the development and evaluation of a comprehensive, practitioner-report measure of practitioners’ and organizational father engagement practices—the *Father Engagement Questionnaire* (FEQ). The internal structure, internal consistency reliability, test–retest stability, and predictive validity of the FEQ were evaluated using a large sample of practitioners involved in delivering parenting interventions. The results provide support for adequate psychometric properties of the FEQ as a research and clinical tool for assessing and monitoring father engagement practices.

The results of the EFA demonstrated that the FEQ measured the five intended content areas as expected: Confidence, Competence, Perceived Effectiveness, Frequency of Strategy Use, and Organizational Practices. Confidence in working with fathers exhibited a strong positive correlation with Competence, suggesting a high degree of construct overlap. This is unsurprising, as both constructs similarly tap practitioners’ self-appraisals of their ability to engage fathers. Nonetheless, the two constructs are not redundant, as the confidence ratings were made with respect to more general aspects of working with fathers (e.g., “communicating with fathers” and “understanding fathers’ needs”), while competence ratings were made with respect to using specific father engagement strategies (e.g., “directly inviting fathers who are reluctant to attend” and “listening to fathers and exploring their barriers to engagement”). Moreover, both Competence and Confidence were moderately and positively correlated with Frequency of Strategy Use. This converges with the theoretical expectation that higher levels of self-efficacy (i.e., seeing oneself as competent) and confidence is likely to be positively associated with actual behavior with respect to using father engagement strategies. Organizational Practices was also moderately and positively correlated with practitioners’ Frequency of Strategy Use, suggesting that organizational practices can facilitate the use of father engagement strategies at the practitioner level, and/or vice versa. Overall, it is recommended that the FEQ is used as a multidimensional measure, assessing related but distinct aspects of father engagement.

All five scales also demonstrated acceptable internal consistency reliabilities and test–retest stability. The test–retest stability for two of the scales—Confidence and Frequency of Strategy Use—while acceptable, were lower than ideal with ICCs of 0.58 and 0.62 respectively. This suggests that the constructs possess a moderate degree of stability. Along with measurement error, this could be attributed to transient error due to random variations in respondents’ psychological states across time (Schmidt et al. 2003). It is also possible that completing the questionnaire at time point one may have prompted practitioners to be more aware of and modify their father engagement practices during the two-week period. Therefore, some degree of caution is needed when interpreting practitioners’ self-assessments and it may be helpful in future research to triangulate the data with more objective measures of competencies such as supervisors’ ratings or observational data on practitioners’ father engagement practice. Nonetheless, the other three scales (Competence, Perceived Effectiveness, and Confidence) demonstrated good to excellent test–retest stabilities, and all the FEQ scales evidenced good internal consistency reliabilities.

In the absence of existing measures of father engagement practice, we were not able to assess construct validity. Instead, we examined predictive validity by looking at whether the FEQ scales would predict father attendance rates—a critical outcome of interest in father engagement research and practice. Practitioner-reported Frequency of Strategy Use and Organizational Practices both uniquely and significantly predicted a greater likelihood that fathers sometimes and often/always attended sessions, compared to never/rarely. Moreover, practitioners who were more confident in working with fathers were also more likely to report fathers often/always (as opposed to never/rarely) attended sessions. These results are consistent with the findings of a practitioner survey, which found that practitioners who reported a combination of high levels of confidence in working with fathers and frequency of using father engagement strategies also reported higher father attendance rates (Tully et al. 2018). Tully et al. found that organizational support was associated with a greater likelihood of father attendance, which converges with the present finding that organizational practices predicted father attendance rates. In sum, it appears that it is the combination of practitioners’ confidence in working with fathers and their own and their services’ frequency of using specific engagement strategies that are the most powerful predictors of father attendance rates.

Unexpectedly, practitioners who provided higher ratings of perceived effectiveness of father engagement strategies were more likely to report that fathers never/rarely, as opposed to sometimes, attend sessions. While this is the opposite of what would be expected, the mean ratings of perceived effectiveness for practitioners who reported fathers never/rarely attend (3.91), fathers sometimes attend (3.79), and fathers often/always attend (3.95) were very similar, with only a slight dip in the middle category. This, along with the notable ceiling effects for perceived effectiveness ratings overall, may have contributed to the unexpected result. However, perceived effectiveness of father engagement strategies may be less important for the prediction of father attendance rates, compared to the other scales in the FEQ such as frequency of strategy use.

### Limitations

There are a number of caveats to the present study. Firstly, there is the need to further establish the construct validity of the FEQ within a nomological network. While the FEQ uniquely assesses father engagement practice in the delivery of parenting interventions, and there are no other existing measures that assess an equivalent construct, it may prove challenging to demonstrate construct validity. However, with increasing research focusing on father engagement, new measures may be developed, and it will be important to examine the convergent and divergent validity of the FEQ scales with measures of other related constructs such as attitudes and beliefs about fathers and the role of fathering, practitioners’ self-efficacy, and organizational culture. Nonetheless, the present study did provide preliminary evidence of predictive validity of the measure in relation to father attendance rates. Secondly, further research is needed to confirm the psychometric properties of the questionnaire (factor structure, internal consistency reliability, and test–retest stability) on separate samples of practitioners and to confirm the factor structure through confirmatory factor analysis and/or Rasch analysis. Most of the practitioners in the present sample self-selected to participate as part of a larger father engagement training study, and thus may have been more motivated towards father-inclusive practice. However, a strength of the present study is the inclusion of a large sample of diverse practitioners (including psychologists, social workers, and other professions) involved in the delivery of parenting interventions. Finally, as one of the key outcomes of father engagement research and training is father attendance rates, future studies would benefit from obtaining objective father attendance rates, such as from case file records. Rates of attendance were self-reported by practitioners in the present study, and this method may be subject to bias.

With these caveats in mind, the present study has reported on the development and psychometric evaluation of a much needed multidimensional measure of father engagement practices—the FEQ—in the context of increasing research and practice attention to promoting father engagement in parenting interventions for child wellbeing. Given the preliminary evidence for its psychometric reliability and validity presented in this study, the FEQ can serve as a useful empirical tool for research, training and practice. Researchers can use the FEQ to assess aspects of practitioners’ father engagement practice such as their confidence, competence and frequency of using father engagement strategies, and how these relate to father engagement, parent, and child outcomes of a parenting intervention; as well as monitor baseline and changes in father engagement practice as a result of any training initiatives to promote father engagement. Likewise, organizations can use the FEQ as an evaluation tool to gather information, understand, and monitor father engagement practices. However, prior to widescale use of the measure, it will be important to further examine the psychometric properties of the scale as well as assess the extent to which the FEQ detects changes in practitioners’ competencies and organizational practices, especially following participation in father engagement training. In the long term, it is hoped that the FEQ will promote father engagement research and practice, with the aim of enhancing the benefits of parenting interventions for child wellbeing.
